# Unveiling viral pathogens in acute respiratory disease: Insights from viral metagenomics in patients from the State of Alagoas, Brazil

**DOI:** 10.1371/journal.pntd.0012536

**Published:** 2024-09-23

**Authors:** Gabriel Montenegro de Campos, Hazerral de Oliveira Santos, Alex Ranieri Jerônimo Lima, Anderson Brandão Leite, Gabriela Ribeiro, Jardelina de Souza Todão Bernardino, Jean Phellipe Marques do Nascimento, Juliana Vanessa Cavalcante Souza, Loyze Paola Oliveira de Lima, Marlon Breno Zampieri Lima, Mykaella Andrade de Araújo, Marta Giovanetti, Esper Georges Kallas, Sandra Coccuzzo Sampaio, Maria Carolina Elias, Svetoslav Nanev Slavov

**Affiliations:** 1 Post-Graduation Program of Clinical Oncology, Stem-Cells and Cell Therapy, Faculty of Medicine of Ribeirão Preto, University of São Paulo, Ribeirão Preto, Brazil; 2 Central Public Health Laboratory (LACEN) of the State of Alagoas, Maceio, Alagoas, Brazil; 3 Center for Viral Surveillance and Serologic Evaluation (CeVIVAS), Butantan Institute, São Paulo, Brazil; 4 Department of Sciences and Technologies for Sustainable Development and One Health, Universita Campus Bio-Medico di Roma, Rome, Italy; 5 Instituto René Rachou, Fundação Oswaldo Cruz, Belo Horizonte, Minas Gerais, Brazil; 6 Climate Amplified Diseases and Epidemics (CLIMADE), Rio de Janeiro, Brazil, United States of America; University of Geneva Hospitals, SWITZERLAND

## Abstract

**Background:**

Respiratory illness affects individuals across all age demographics on a global scale, often precipitated by viral infections. The symptomatic manifestations of these diseases bear clinical resemblance, complicating the accurate determination of their etiological origins. Furthermore, the diagnostic panels for respiratory pathogens used within local medical practices, may not encompass the full spectrum of viral agents responsible for such ailments. Consequently, a significant number of clinically important viral pathogens may remain undetected.

**Methods and findings:**

In the light of this, we conducted a metagenomic examination of 66 nasopharyngeal swab specimens, obtained from patients presenting with acute respiratory conditions yet tested negative by the standard diagnostic panels available locally. These specimens were obtained from the Public Health Laboratory, Maceio, State of Alagoas. Our findings indicate a predominant diagnostic escape of rhinoviruses and notably enterovirus D68. Moreover, our study identified a substantial quantity of sequence reads attributed to human respirovirus 3 (human parainfluenza 3) along with various herpresviruses including human herpesvirus-1, Epstein-Barr virus (Human herpesvirus-4), Human herpesviruses 6 and 7 and human parvovirus B19 (B19V). Notably, the metagenomic analysis uncovered a widespread presence of the emerging human vientovirus FB in most of sample pools, though its clinical importance remains to be elucidated.

**Conclusions:**

The obtained results in this study underscore the invaluable role of viral metagenomics in the identification of underrecognized viruses bearing clinical relevance. Furthermore, it offers insights into the dissemination of these pathogens within the studied area, thereby informing public health strategies aimed at enhancing diagnostic accuracy and improving patient care.

## Author summary

The emergence of SARS-CoV-2 has reshaped our understanding of respiratory viruses, highlighting the challenges in etiological diagnosis due to symptom similarities. Metagenomics, an innovative approach, has revolutionized respiratory infection diagnosis by unbiasedly sequencing all genetic material in a sample, enabling the identification of previously unknown or unsuspected viruses where traditional methods often fail. In a study conducted in Alagoas, a Northeast Brazilian state with low human development indexes, samples obtained from patients with flu-like symptoms negative for locally applied diagnostic kits revealed diagnostic escape of rhinoviruses and a notably prevalent enterovirus D68. Additionally, substantial reads attributed to human respirovirus 3 (parainfluenza virus 3) and various herpesviruses, including human herpesvirus-1, Epstein-Barr virus, human herpesviruses 6 and 7, and human parvovirus B19, were identified. These findings are pivotal for differentiating viruses not detected by routinely implemented diagnostic procedures. Metagenomics shows great promise in diagnosing and surveilling respiratory viruses, providing insights into their epidemiology, evolution, and public health impact.

## Introduction

Acute respiratory illness represents the most common cause of morbidity and mortality across all age demographics worldwide, with its impact being particularly pronounced among children and the elderly [1]. The process of etiological diagnosis of respiratory infections is challenging, primarily due to the vast diversity of viruses that potentially cause similar clinical symptoms. Notably, orthomyxoviruses, paramyxoviruses, picornaviruses, picornaviruses, coronaviruses, and adenoviruses might be implicated in these symptoms [2]. Complicating matters further include emerging respiratory viruses that remain largely undetectable through contemporary diagnostic platforms.

Respiratory PCR viral panels have emerged as a potent tool for diagnosing viral respiratory infections, offering the capability to identify multiple viruses with remarkable sensitivity [3]. Nevertheless, the efficiency of these molecular assays is hampered by various limitations, including the exclusive detection of specific gene subsets and primer mismatches coupled with the extensive heterogeneity of respiratory viruses. This scenario renders occasionally the etiological agents of such diseases undiagnosed. For instance, human rhinoviruses belonging to the *Picornaviridae* family and recognized a leading cause of acute upper respiratory tract diseases, comprise over 100 types, making their diagnosis via single molecular tests a significant challenge [4]. Consequently, the unbiased nature of next-generation sequencing and viral metagenomics has been increasingly harnessed for diagnosing respiratory infections [5] and identifying emerging respiratory viruses [6].

In our study, we applied viral metagenomics to identify respiratory viruses that were not detected by the routine respiratory virus employed at the Public Health Laboratory in Maceio, State of Alagoas, Brazil.

## Methods

### Ethics statement

Ethical approval for this study was obtained from the Ethical Committee of the Faculty of Medicine of São Paulo, University of São Paulo (Project Number: CAAE 68586623.0.0000.0068). Due to the reuse of clinical samples originally obtained for a larger project, the anonymous nature of this survey, and the formation of unidentified sample pools where participant identification was not required, the Institutional Ethics Committee granted a waiver of informed consent.

### Clinical samples

**Fig 1:**
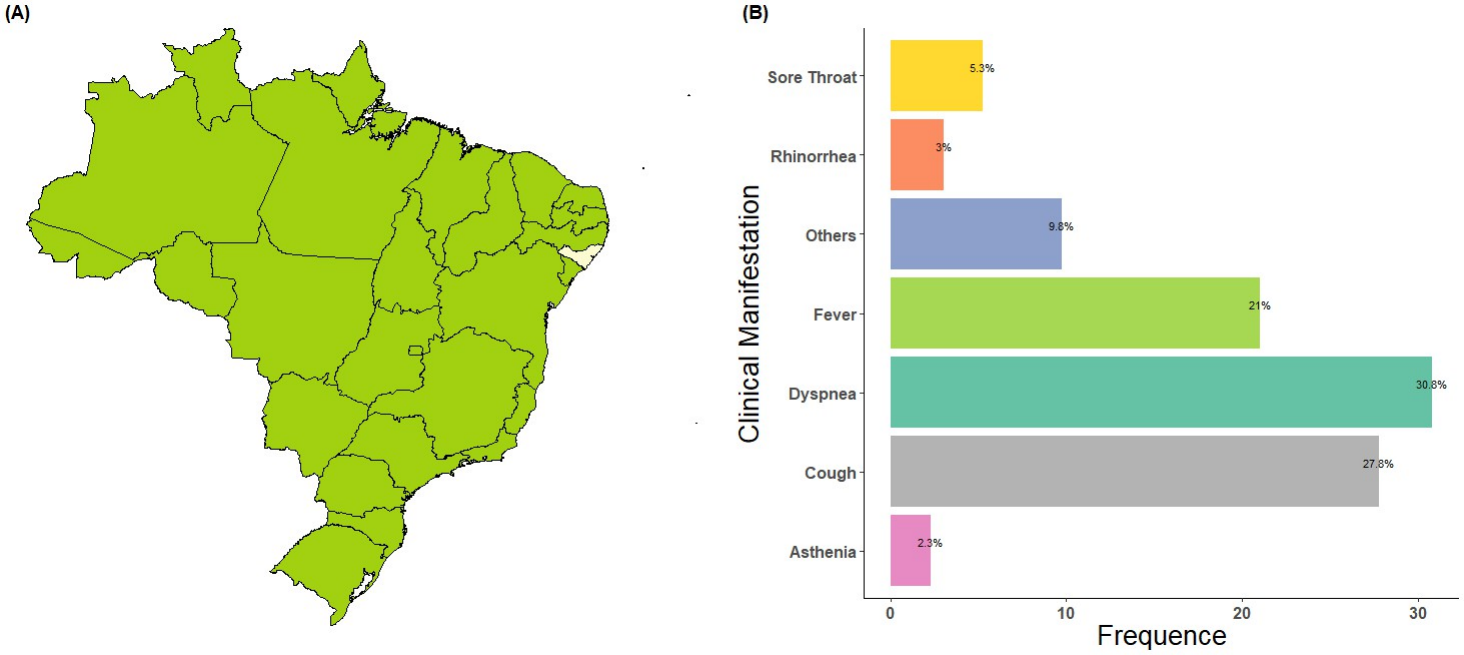
Spatial location of the study area and clinical symptoms of the studied group. A. Map of Brazil illustrating the geographic localization of the State of Alagoas. B. Main clinical manifestations reported by patients. The base map of Brazil was sourced from Natural Earth and can be accessed at Natural Earth data (https://www.naturalearthdata.com/downloads/10m-cultural-vectors/10m-admin-1-states-provinces). The geographic map was edited in R using the rnaturalearth package, v. 1.0.1 (https://cran.r-project.org/web/packages/rnaturalearth/index.html).

In this study, we retrospectively submitted 66 nasopharyngeal swabs to metagenomic analysis. These swabs were received for etiological diagnosis at the Central Public Health Laboratory of Alagoas between May and September 2023. The samples were obtained from hospitalized or ambulatory patients exhibiting upper or lower respiratory symptoms and had tested negative for respiratory viruses, including SARS-CoV-2, Influenza A/B, Respiratory Syncytial Virus, Adenovirus, Metapneumovirus, and Rhinovirus/Enterovirus. The routine testing was conducted using the respiratory virus panel pipeline kit, which includes the fiveplex kit for SARS-CoV-2/Influenza A/Influenza B/Metapneumovirus/RNase P and the fourplex kit for Respiratory Syncytial Virus/Adenovirus/Rhinovirus/RNase P (Bio-Manguinhos, Rio de Janeiro, Brazil). The samples were collected from individuals residing in 27 different cities of the State of Alagoas, covering the most important geographical regions of this Brazilian state (Fig 1A and S1 Table). Such a strategy provides a comprehensive overview of undetected viral agents potentially involved in acute respiratory diseases. Municipalities adhere to stringent protocol for sample collection and while regional differences may exist, the sampling conditions were uniform minimizing the risk of bias.

### Sample preparation and Next-Generation Sequencing

Following vortexing, 600 μL of individual nasopharyngeal swab samples underwent pre-treatment with Turbo DNase (ThermoFisher Scientific, Waltham, MA, USA) to remove host/bacterial DNA. After DNase inactivation, 6 to 8 individual nasopharyngeal samples were randomly pooled in order to minimize sequencing costs and proportionally increase the number of tested samples. Viral nucleic acids were extracted from the pooled samples using the High Pure Viral Nucleic Acid Large Volume Kit (Roche, Basel, Switzerland) with minor modifications, including the use of GenElute Linear Polyacrylamide carrier (LPA) (Merck, Darmstadt, Germany) for nucleic acid concentration and isopropanol for precipitation. Five microliters of extracted nucleic acids underwent reverse transcription using the Superscript III First-Strand Synthesis System (ThermoFisher Scientific, Waltham, MA, USA). cDNA amplification was then performed using the QuantiTect Whole Transcriptome Kit (QIAGEN, Hilden, Germany) with high-yield isothermal amplification at 30 °C for 8 h. Sequence libraries were prepared using the DNA Prep Library Preparation Kit (Illumina, San Diego, CA, USA) and Nextera DNA CD Indexes (Illumina, San Diego, CA, USA). Paired-end sequencing of the dual-indexed libraries was performed by Illumina NextSeq 2000 sequencing platform using the NextSeq P3 flowcell (300 cycles) (Illumina, San Diego, CA, USA), following the manufacturer’s instructions.

### Bioinformatic analysis and virus annotation

Raw sequence data (in fastq format) underwent quality control inspection using FastQC v0.12.1 [7] following filtering and adapter removal to obtain high-quality reads (phred quality score over 30) using Fastp v.0.20.0 [8]. Additionally, Fastp was utilized for trimming PolyX (3′) and PolyG tails, as well as removal of duplicate reads.

To isolate viral reads, high-quality reads were first mapped to the *Homo sapiens* reference genome (NCBI GRCh38.p14 - updated on February 3, 2022) and taxonomic classification of non-human reads was performed. Mapping was performed BWA 0.7.17-r1188 software [9] using BWA-MEM (Burrows-Wheeler Aligner—Maximal Exact Matches) algorithm [10]. Unmapped (non-human) reads were extracted using samtools v.1.12 [11] and submitted to Kraken2 v 2.1.3 [12] for taxonomic classification and virome inference. Kraken2 utilized the RefSeq complete viral genomes/proteins database (last updated on February 06, 2024).

To visually represent viral abundance, bar plots were generated using the R programming language v. 4.3.1 — "Beagle Scouts" (R Core Team, 2024), in conjunction with the integrated development environment (IDE) RStudio 2023.06.0+421 "Mountain Hydrangea" (RStudio Team, 2024). Key libraries utilized for data analysis included: readr v.2.1.4 [13], tidyverse v.2.0.0 [14] for data analysis, ggplot2 v.3.4.4 [15] for graphic visualization, and ggsci v. 3.0.0 [16] for color schemes.

## Results

The majority of samples were obtained from individuals diagnosed with lower respiratory tract symptoms (84.8%), while the rest presented with upper respiratory tract symptoms (15.2%). The most common clinical manifestations are depicted in Fig 1B. The study group had an average age of 43.14 years (interquartile range [IQR]: 9.00–71.75), with 34 females and 32 males. In terms of age distribution, 31.8% of the included samples belonged to children (aged 1 month to 18 years) and 68.2% to adults (aged 18–95 years). Among the underlying diseases, the most common was diabetes mellitus (n = 14/66, 21.2%), followed by chronic heart disease (n = 7/66, 10.6%), oncologic diseases (n = 3/66, 4.5%), obesity (n = 2/66, 3.0%), and asthma (n = 1/66, 1.5%).

The obtained sequence data was deemed sufficient for robust metagenomic analysis. The quantitative characteristics of the NGS data, post-read trimming, extraction of non-viral reads, and classification of viral reads by Kraken2, are summarized in Table 1. Across all pools, the median proportion of viral reads accounted for 9.1% of the total number of unmapped (non-human) reads.

**Table 1:** Viral read quantity following quality control and mapping in the tested samples Pools.

Pool Number	Total Number of Reads (Millions)	Number of Reads after Filtering and Trimming (Millions)	Unmapped Reads (Millions)	Viral Reads*
1	94.1	69.8	1.9	186,999 (9.64%)
2	96.0	67.0	14.0	3,302,451 (23.67%)
3	158.2	119.2	25.4	271,332 (1.07%)
4	65.8	54.6	2.5	375,644 (14.95%)
5	102.2	81.9	1.9	31,908 (1.65%)
6	139.0	105.2	4.2	53,937 (1.29%)
7	107.6	83.7	2.5	496,628 (20.11%)
8	70.6	54.1	0.8	65,155 (7.77%)
9	88.4	70.6	1.9	201,032 (10.64%)
10	6.7	5.7	0.1	10,547 (16.08%)

*Percentage of viral reads calculated relative to unmapped reads following quality control and mapping of tested pools

A notable finding was the detection of numerous clinically significant viruses that were missed by the routinely used diagnostic assays. To exclude the presence of contaminating sequences, we performed mapping of the reads of clinically important viruses by pool. We observed a random distribution of the reads across the viral reference genomes, within distantly located genomic regions which is an indication of absence of contamination. Due to the pooling strategy used in our study, we did not conduct more in-depth investigations of the obtained genomes, including phylogenetic analysis. The mapping of the reads of clinically significant viruses identified by pool is shown in S1 Fig.

**Fig 2:**
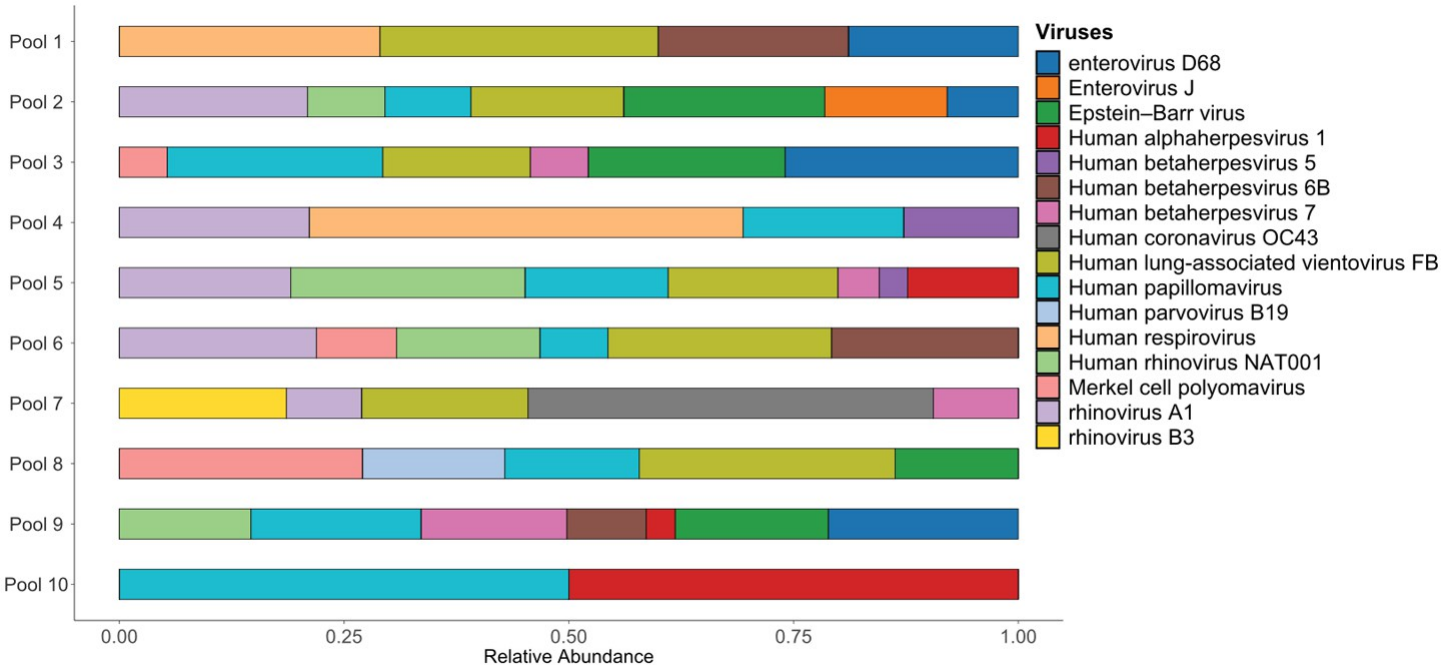
Horizontal bar plot of the abundance of viruses with clinical importance identified among patients from the State of Alagoas who showed negative results by the locally applied respiratory virus panels. The horizontal axis of the plot (X-axis) indicates the relative abundance of the viral reads per pool with values ranging from 0.00 (0%) to 1 (100%). Y-axis denotes the pool number.

The most abundant viruses across all pools belonged to the *Picornaviridae* family (Genus *Enterovirus*), particularly rhinoviruses and enteroviruses. Rhinovirus A (A1) and Human rhinovirus B1 were the most abundant representatives of Rhinoviruses, while Enterovirus D68 predominated among Enteroviruses, with sporadic identification of enteroviruses of the J group (Fig 2). Following Picornaviruses, viruses associated with systemic and latent infections, such as human herpesviruses and human parvovirus B19 (B19V), were detected. Epstein Barr virus (EBV) was the most prevalent human herpesvirus, particularly prominent in pool 2. Human herpesvirus 6 and Human herpesvirus 7 were also identified, albeit at lower levels. B19V was detected in pool 8, a common causative agent of erythema infectiosum with significant morbidity among children. Additionally, human respirovirus 3 (human parainfluenza virus type 3) belonging to the *Paramyxoviridae* family and known to cause acute respiratory infections, was abundant, particularly in pool 4 (Fig 2). An intriguing observation was the detection of the emerging circular virus Human-lung associated vientovirus FB in 7 of the tested pools (70%), with particularly high read counts observed in pools 1, 2 and 6 (Fig 2).

**Fig 3:**
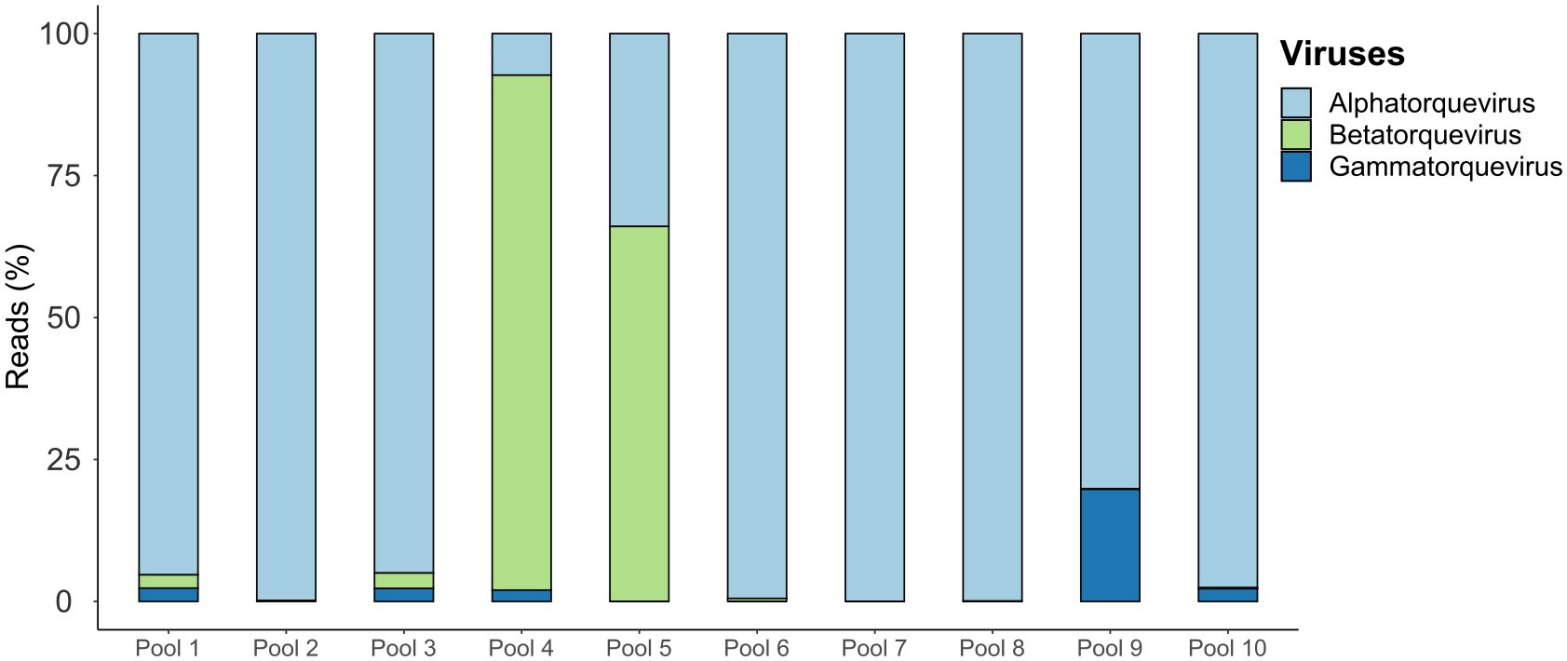
Bar plot graphics representing the abundance of the detected commensal viruses. Bar plot of the relative abundance of the commensal viruses (anelloviruses) detected at genus level. X axis: Pool Number. Y-axis: percentage of reads referring to each Anellovirus genus.

Commensal viruses, which are part of the normal human virome, were also identified, including representatives of the *Anelloviridae* family (Genus Alphatorquevirus-Torque Teno virus (TTVs), Betatorquevirus- Torque teno mini virus (TTMvs), and Gammatorquevirus- Torque teno midi virus (TTMDVs), as well as bacteriophages. Notably, in Pool 10, 13.07% of all viral reads belonged to the *Anelloviridae* family. The majority of anellovirus reads were classified as belonging to the *Alphatorquevirus* genus (Fig 3).

## Discussion

In this study, we conducted viral metagenomics analysis on samples obtained from individuals with acute respiratory disease in Brazilian state of Alagoas, who tested negative for locally used respiratory virus panels. Through this approach, we identified underscored viral pathogens that could be implicated in acute flu-like respiratory disease, aiding in establishing accurate differential diagnoses and improving molecular diagnosis of viral diseases in Brazil.

The tested samples exhibited the presence of several viruses with significant clinical impact, potentially directly involved in acute respiratory and systemic infections. The most prevalent viral species that were undiagnosed were enteroviruses, particularly rhinoviruses (A1, B1, C1, NAT001) and enterovirus types D68 and J. Many infections caused by the representatives of the *Enterovirus* genus remain undiagnosed and ungenotyped, highlighting the importance of understanding the circulating of specific enterovirus types to implement strategies for reducing associated morbidity and mortality [17]. An inherent challenge in diagnosing enteroviruses lies in their remarkable diversity, with rhinoviruses alone comprising three species and over 169 subtypes. This vast diversification necessitates initial amplification of conserved viral regions, followed by genotyping through sequencing methods [17]. Although the currently used detection techniques for enterovirus diagnosis are designed to target conserved viral regions, such assays may detect enterovirus types with varying sensitivity. For instance, Enterovirus D68 has been detected only at higher copy numbers [18]. This variability in sensitivity might be one of the reasons for a negative result obtained for the rhinovirus/enterovirus testing protocol of the respective Public Health Laboratory. A suitable strategy to investigate why the rhinovirus detection system does not amplify enterovirus D68 and several rhinoviruses is to align the primer sequences used with reference genomes in the search for primer/probe binding mutations. However, such an analysis was not possible as the primer sequences are proprietary information protected by the manufacturer’s patent. Additionally, we assembled the samples in pools, and when there is more than one positive sample in a pool, it might lead to the assembly of chimeric sequences with erroneously distributed mutations that might not be significant. Consequently, metagenomic approaches are gaining traction for enterovirus identification, particularly in cases involving severe neurological complications [19].

In four pools (1, 2, 3 and 9), we detected the presence of enterovirus D68, an emerging pathogen known to cause acute respiratory disease, primarily affecting pediatric patients and potentially leading to complications such as flaccid myelitis [20]. In Brazil, there exists limited data concerning the dissemination of enterovirus D68, with several studies reporting isolated virus detection in several Brazilian regions [21–23]. Furthermore, complete genomes of this virus isolated from Brazil and South America are currently unavailable. Our findings and the frequency of detection of this virus suggest its presence in the State of Alagoas, possibly indicating an outbreak. Further studies, including molecular genotyping, are urgently required to investigate Enterovirus D68 prevalence, genotypes, and clinical impact in the State of Alagoas and Brazil as a whole.

In two pools (1 and 4), we detected Human respirovirus 3 in a substantial number of reads. This virus, belonging to *Respirovirus* genus, is a prevalent cause of bronchiolitis in pediatric patients, second only to Respiratory syncytial virus [24,25]. Several studies describe the circulation of respiroviruses in Brazil especially in South and Southeast Brazil [26,27]. However, there is scarce information for the dissemination of this agent in Northeast Brazil. In that respect, our study brings important information for the circulation of this virus in that region. Consequently, the inclusion of respirovirus surveillance and testing in diagnostic kits for Public Laboratories could facilitate accurate diagnosis of this viral infection. Furthermore, we identified coronavirus OC43 in pool 7, which is a common respiratory pathogen but was also not included in the respiratory virus panel used in this location [28]. Metagenomic sequencing shows high cost to be applied for routine diagnosis of respiratory viruses and requires sophisticated bioinformatic analysis infrastructure. Therefore, our survey that was conducted to identify undetected viruses might be pivotal for updating respiratory panels used for routine diagnosis in Brazil.

We also identified viruses associated with latent infections, predominantly belonging to the Herpesviridae family, including Human herpes simplex virus 1 (HSV-1), EBV, Human herpesvirus 4, Human herpesvirus 6, Human herpesvirus 7. Human herpesvirus infections are widely distributed in the human population and can lead to severe clinical consequences in states of immune suppression. It is challenging to attribute the identification of herpesviruses to observed clinical manifestations primarily because of low-level shedding may occur in healthy individuals [29,30]. EBV is known to cause acute infection termed infectious mononucleosis, which is common among the pediatric population. Clinical manifestations typically include fever, pharyngitis, cervical lymphadenopathy, and lymphocytosis. Although we detected a high read number belonging to EBV in one of the pools, which might indicate a high copy number, further estimations of the viral load are necessary to confirm if this detection was related to an acute infection. Human herpesvirus type 6, which also causes the acute infection known as exanthema subitum, characterized by fever and rash might be asymptomatically excreted in saliva [30]. Additional investigations are needed to correlate this finding with clinical manifestations.

On the other hand, B19V causes erythema infectiosum, also known as “fifth disease”, a pediatric infection characterized by malaise, fever, and facial maculopapular rash [31]. Diagnosing B19V can be challenging, as it is not typically included in respiratory virus panels and is only tested when there is suspicion based on the presence of typical symptoms. Consequently, metagenomics can be useful for identifying B19V circulation in cases of acute respiratory disease. Performing viral metagenomics analysis on negative samples not only provides insight into the viral landscape associated with flu-like diseases but also aids healthcare providers in making adequate differential diagnoses based on information of the locally circulating virus agents.

Interestingly in a significant percentage of the tested pools (n = 7/10, 70%), we identified the human vientovirus FB. This emerging respiratory virus, discovered in 2019, belongs to the *Redondoviridae* family. While these viruses have been associated with various clinical conditions [32] their global distribution suggests that they may be highly prevalent and potentially be considered as commensal agents [33]. Based on the results of our study, which showed a high detection frequency of vientovirus FB and varying numbers of sequence reads (141–31,968), we also adhere to the hypothesis that it might be a commensal virus. Additionally, this is the first instance of detecting such an agent in this Brazilian location. Therefore, further studies and more detailed investigations are necessary to estimate its prevalence among healthy individuals and patients with respiratory diseases. This will provide further insights into whether it is associated with respiratory symptomatology or merely a bystander, forming part of the normal human respirome.

Our study has several limitations. It is important to note that metagenomics detects the abundance of all viruses in a given sample, including commensal viruses with unknown or no clinical relevance, as well as those that are clinically significant. Consequently, the genomic data obtained from metagenomic sequencing must be carefully interpreted. Clinically suspicious viruses should be confirmed through molecular quantification techniques and classic virological methods such as viral culture. Additionally, metagenomics identifies only viral genetic sequences, which may not necessarily correspond to infectious viral particles. Our study also analyzed pooled samples rather than individual ones, preventing the establishment of a relationship between the identified viruses and clinical symptomatology.

In conclusion, our study highlights the potential of metagenomics in identifying viral respiratory agents in samples that have shown negative result with the locally used routine diagnostic kits. The obtained results provide valuable information for public health policies regarding the dissemination of undetected agents in this region. Additionally, our findings suggest that viral metagenomics might contribute to significant improvements of the viral diagnostic panels which can enhance the control and surveillance of acute flu-like diseases.

## Supporting information

S1 Fig Mapping of the obtained reads of clinically important viruses per pool (showed by the virus name) across the reference genomes. This was performed in order to show the read distribution and absence of contamination.
(TIF)

S1 Table Municipalities in the State of Alagoas from Which Clinical Samples Were Obtained and the Corresponding Number of Samples. (DOCX)
