# Supplementary figures and images for: Unveiling viral pathogens in acute respiratory disease: Insights from viral metagenomics in patients from the State of Alagoas, Brazil
Source: PLoS Negl Trop Dis. 2024 Sep 23;18(9):e0012536. doi: 10.1371/journal.pntd.0012536 (PMC11460670; doi:10.1371/journal.pntd.0012536)

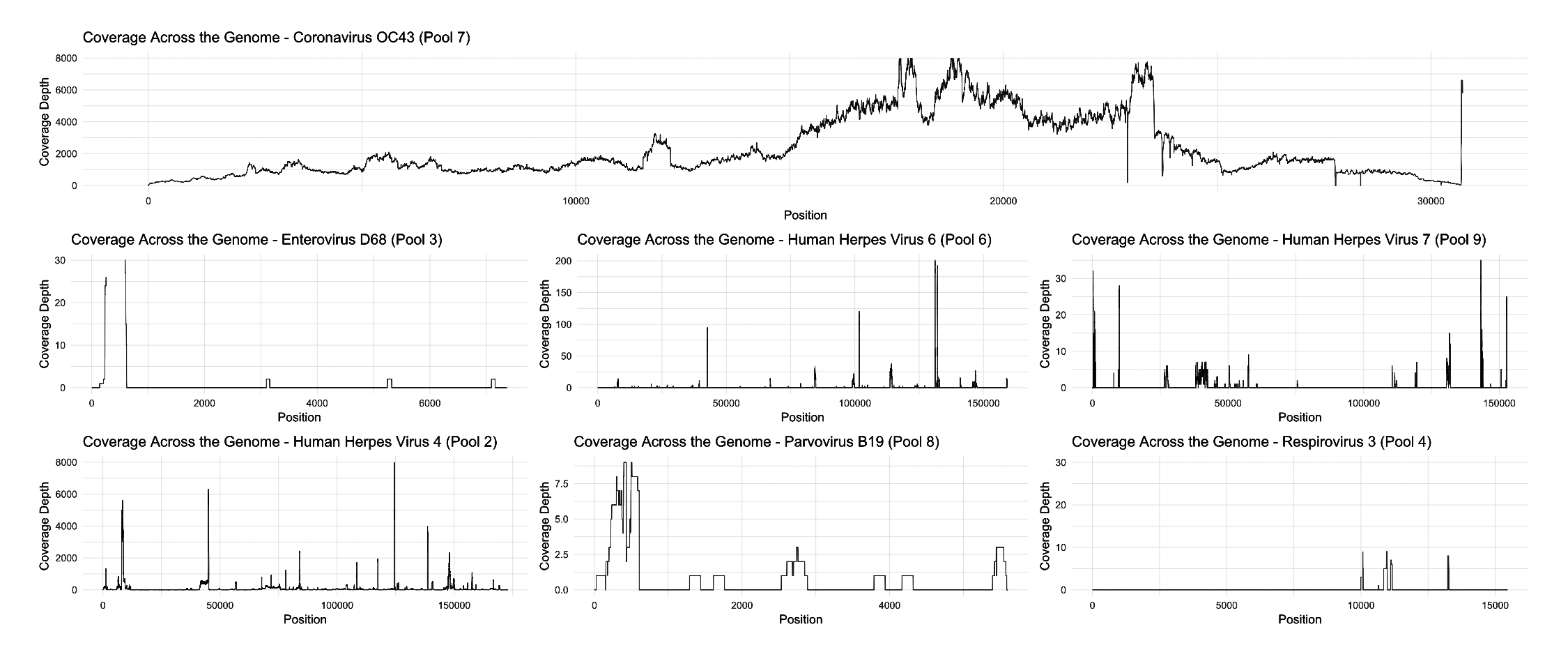

Supplement: S1 Fig — This was performed in order to show the read distribution and absence of contamination. (TIF) [file pntd.0012536.s001.tif]
