# Supplementary material for: Unveiling viral pathogens in acute respiratory disease: Insights from viral metagenomics in patients from the State of Alagoas, Brazil
Source: PLoS Negl Trop Dis. 2024 Sep 23;18(9):e0012536. doi: 10.1371/journal.pntd.0012536 (PMC11460670; doi:10.1371/journal.pntd.0012536)
Supplement: S1 Table — (DOCX) [file pntd.0012536.s002.docx]

| **State of Alagoas** | |
| --- | --- |
| **Municipality name** | **Number of samples obtained** |
| Maceió | 23 |
| Rio Largo | 3 |
| União dos Palmares | 1 |
| Arapiraca | 8 |
| Pariconha | 1 |
| Delmiro Gouveia | 4 |
| Estrela de Alagoas | 1 |
| Girau do Ponciano | 3 |
| Satuba | 2 |
| Mata Grande | 1 |
| Piranhas | 1 |
| Olho da Água do Casado | 1 |
| São José da Laje | 2 |
| Major Isidoro | 1 |
| São Miguel dos Campos | 2 |
| Pilar | 1 |
| São Luís do Quitunde | 1 |
| Messias | 1 |
| Marechal Deodoro | 1 |
| Inhapi | 1 |
| Igaci | 1 |
| Maragogi | 1 |
| Campo Grande | 1 |
| Junqueiro | 1 |
| Andaia | 1 |
| Boca da Mata | 1 |
| Palmeira dos Índios | 1 |
